# A single-camera gaze tracking system under natural light

**DOI:** 10.16910/jemr.11.4.5

**Published:** 2018-10-20

**Authors:** Feng Xiao, Dandan Zheng, Kejie Huang, Yue Qiu, Haibin Shen

**Affiliations:** Institute of VLSI Design, Zhejiang University, China

**Keywords:** Eye movement, eye tracking, gaze, usability, single-camera, facial landmark, iris center, anchor point, head pose, mapping functions

## Abstract

Gaze tracking is a human-computer interaction technology, and it has been widely studied in the academic and industrial fields. However, constrained by the performance of the specific sensors and algorithms, it has not been popularized for everyone. This paper proposes a single-camera gaze tracking system under natural light to enable its versatility. The iris center and anchor point are the most crucial factors for the accuracy of the system. The accurate iris center is detected by the simple active contour snakuscule, which is initialized by the prior knowledge of eye anatomical dimensions. After that, a novel anchor point is computed by the stable facial landmarks. Next, second-order mapping functions use the eye vectors and the head pose to estimate the points of regard. Finally, the gaze errors are improved by implementing a weight coefficient on the points of regard of the left and right eyes. The feature position of the iris center achieves an accuracy of 98.87% on the GI4E database when the normalized error is lower than 0.05. The accuracy of the gaze tracking method is superior to the-state-of-the-art appearance-based and feature- based methods on the EYEDIAP database.

## Introduction

Gaze tracking is a kind of human-computer interaction technology that creates an easy and effective interaction for serving the disabled, learning, entertainment, etc. Meanwhile, it is also a research tool, and it has been widely used in marketing studies (1), reading research (2), and so forth. Gaze tracking techniques can be divided into electrooculography-based, coils-based, and video-based (infrared and natural light) techniques and so on (3). The third technique is less intrusive than the first two, which require physical contact sensors such as electrodes and scleral coils.

Today, a variety of existing remote video-based gaze tracking systems under infrared (IR) light in academia and industry have achieved accurate results. For instance, the Dual-Purkinje-Image (DPI) gaze tracker (4) achieves an accuracy better than 0.1° (5). The Eyelink 1000 system performs at an accuracy below 0.5° with a white background (6). However, infrared sources are sensitive to ambient light. IR gaze trackers also have reflection problems when people wear glasses. Therefore, development of a gaze tracking system under natural light has become an increasingly important field of research.

Recently, video-based gaze tracking system under natural light are capable of tracking the gaze. However, some of them rely on multiple cameras (7), High-Definition (HD) cameras (8) and RGB-D cameras (9, 10), which limit their applications. With the popularity of cameras, gaze tracking with a single-camera under natural light becomes a research hot spot, but one of the major challenges is the requirement for an accurate gaze tracking algorithm. Therefore, we concentrate on a regression-based gaze tracking system with a single-camera under natural light in this paper.

The accuracy of regression-based gaze tracking is directly influenced by the eye vectors that are derived from the iris centers and the facial stable point (anchor point or reference point). However, the performance of various iris/pupil center localization methods significantly degrades in low resolution images because of interference such as glass/iris reflection, and eyelid. In addition, the anchor points of the eye corners vibrate with eye rotation (11) and can be blocked due to large head movements. Therefore, an accurate localization method in low resolution images (12) is improved to detect the iris center. Then, a novel anchor point is proposed to overcome the drawbacks mentioned above. Finally, the Points of Regard (POR) of the left and right eyes are combined to improve the accuracy of the system. Compared with other regression-based methods, the main contributions in this paper are listed in the following:

(1) An accurate feature position localization method for the iris center is implemented in low resolution images by combining facial landmarks, the prior knowledge of eye anatomical dimensions, and the simple active contour snakuscule(13).

(2) A novel anchor point is computed by averaging the stable facial landmarks, which improves the accuracy of the gaze tracking system.

(3) A weight coefficient is used on the POR of the left and right eyes to revise the final POR, which reduces the error of the gaze tracking.

The rest of the paper is structured as follows: In the next section, the related work is presented. The details of the proposed method are covered in the *Methods* section. The evaluation of the proposed scheme and statistical results on public databases are shown in the *Evaluation* section. The discussion is presented in the final section.

### Related work

This section overviews gaze tracking systems under natural light. The systems can be classified into feature-based and appearance-based methods (14).

### Feature-based methods

Feature-based methods extract features such as the iris/pupil center, eye corners and iris/pupil contours. Then, model-based and regression-based methods use the features to track the gaze. Model-based methods (10, 15) use a geometric eye model to compute the gaze direction from the features. Regression-based methods (16, 17) compute a mapping function between the gaze direction and eye vectors.

The performance of model-based methods relies on the accurate detection of the iris center. In (10), the iris center was obtained by an ellipse fitting algorithm, where the ellipse of the iris in the image was described by the yaw and pitch angles. J.Li and S.Li (10) achieved 7.6° and 6.7° in horizontal and vertical directions on the public EYEDIAP database (18) with an execution speed of 3 frames per second (fps) on a 2.5-GHz Inter(R) Core(TM) i5-2400S processor. In (15), the shape of the iris was estimated by ellipse fitting. Then, an accuracy of 7° of the gaze direction was inferred by the hypothesis that the shape of the iris appears to deform from circular to elliptical when the iris orientation changes. Wood and Bulling (15) achieved an execution speed of 12 fps on a commodity tablet computer with a quad-core 2 GHz processor. Ellipse fitting has a low consistency and reliability because iris edges or points cannot be accurately extracted in low resolution images.

In addition to the iris center, the anchor point is one of the key features influencing the accuracy of the regression-based methods. In (16), the eye corner was used as the anchor point. Instead of detecting the eye corners, the anchor point in (17) was set as the center coordinate of the patch which contains the inner eye corners and eyebrow edges. The proposed system yielded a mean accuracy of 2.33° and 1.8° in the horizontal and vertical directions on their self-built database and 7.53° on the public UulmHPG database (19). However, eye corners or the center of the patch cannot be accurately detected in low resolution images with a large head rotation.

### Appearance-based methods

Appearance-based methods do not extract specific features and usually learn a mapping function from eye images to gaze directions. In (20), gaze estimation was learned by random regression forests with a significantly larger dataset, which reduced the error by 50% from the work in (21) with an error larger than 10°. In (9), k-nearest neighbor regression and adaptive linear regression were used to learn mapping functions between eye images and gaze directions, which achieved a mean accuracy of 7.2° (keeping the head still) and 8.9° (head movement) on the EYEDIAP database. With the development of deep learning, convolutional neural networks (CNNs) have been used to estimate the gaze with millions of eye images in (22). They proved that a largescale dataset and a large variety of data could improve the accuracy of the appearance-based model for gaze tracking, which achieved errors of 1.71 cm and 2.53 cm without calibration on mobile phones and tablets, respectively. Krafka, et al. (22) achieved a detection rate of 10–15 fps on a typical mobile device. One of the main drawbacks to appearance-based methods is that the appearance of the eyes is significantly affected by the head pose (17). In addition, compared with feature-based methods, appearance-based methods generally require larger numbers of training images.

## Methods

**Figure. 1:**
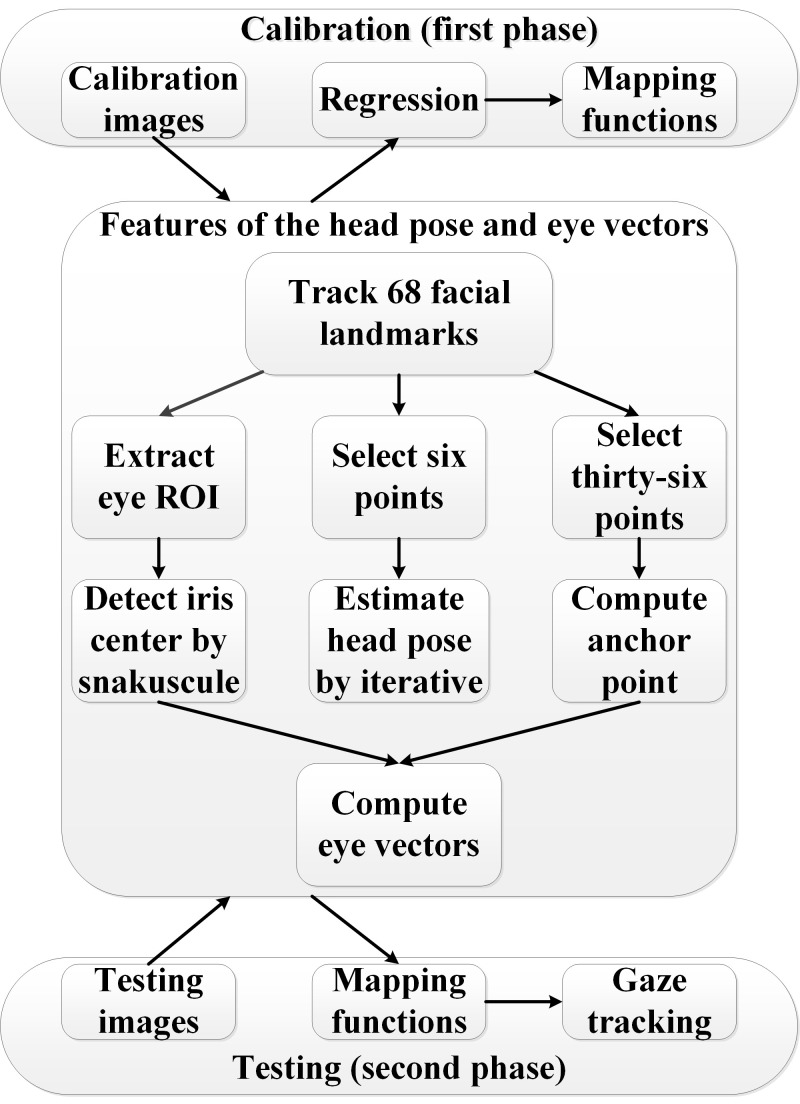
Flow chart of the gaze tracking system

The flow chart of the gaze tracking system is depicted in Figure 1. The system includes calibration and testing phases. In the calibration phase, mapping functions are regressed by the head pose, eye vectors and gaze directions. Afterwards, the head pose, eye vectors and regressive mapping functions are used to track the gaze in the testing phase. The feature extraction consists of three parts for the iris centers, anchor point and head pose calculations.

First, the eye Region of Interests (ROIs) are extracted by twelve points around the eyes, which are tracked by the facial landmarks algorithm in (23). The eye ROIs are resized to twice the original sizes. Then, greyscale erosion is applied. After that, the snakuscule is used to locate the iris centers.

Second, thirty-six stable facial landmarks are used to compute the anchor point. Thereafter, the left and right eye vectors are computed by the iris centers and the anchor point, respectively.

Third, the head pose is estimated based on the six facial landmarks of eye corners, nose tip, mouth corners and chin by using the OpenCV (24) iterative algorithm.

Details of the overall system are discussed in the following subsections.

### Iris center localization

**Figure. 2:**
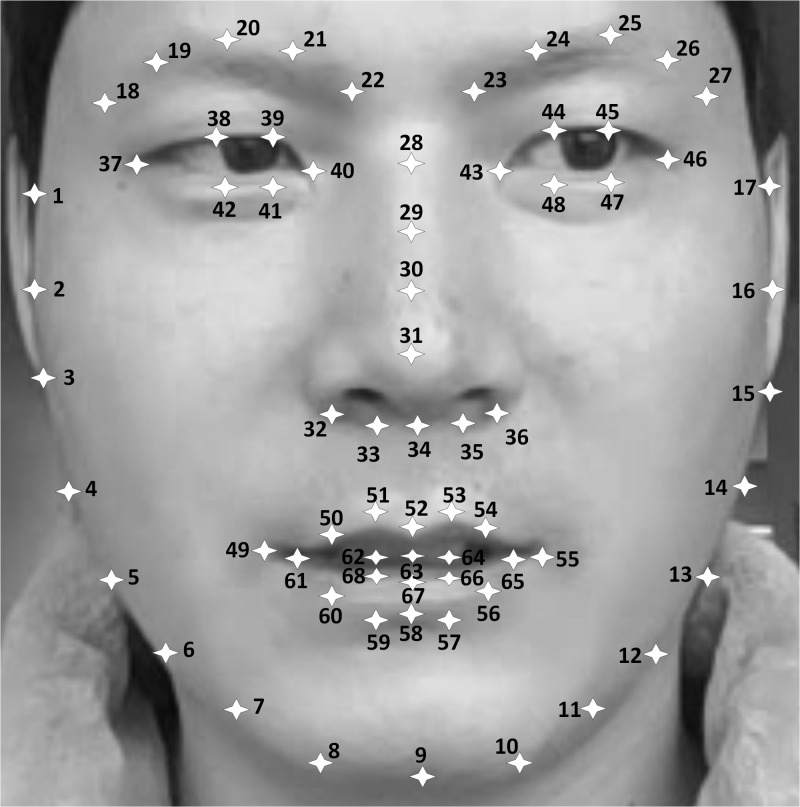
The locations of 68 facial landmarks

The eye ROI should be detected before locating the iris center. Facial landmarks can provide more precise positioning of the mouth, eyes, nose, etc. Therefore, an ensemble of regression trees algorithm (23) is used to detect 68 facial landmarks. This algorithm uses intensity differences between pixels to estimate the positions of 68 facial landmarks. The locations of 68 facial landmarks are shown in Figure 2.

The rectangular eye ROIs are next extracted by the twelve points around the eyes. The boundary coordinates of the eye ROIs are computed by the equations in Table 1.

**Table 1.** The boundary coordinates of eye ROIs.

Left eye	Right eye
*X_l_ = P_43x_*	*X_l_ = P_37x_*
*X_r_ = P_46x_*	*X_r_ = P_40x_*
*Y_t_ = min{P_44y_ ,P_45y_}-3*	*Y_t_ = min{P_38y_ ,P_39y_}-3*
*Y_b_ = max{P_47y_ ,P_48y_}+3*	*Y_b_ = max{P_41y_ ,P_42y_}+3*

Note: X_l_ , X_r_ , Y_t_ and Y_b_ are the left, right, top and bottom coordinates of the eye ROIs. P_ix_ and P_iy_ are respectively the x and y coordinates of the i^th^ facial landmark. max{,} and min{,} denote taking the maximum and minimum values respectively among the two values. The coordinate origin is in the top left corner of the image.

**Figure. 3:**
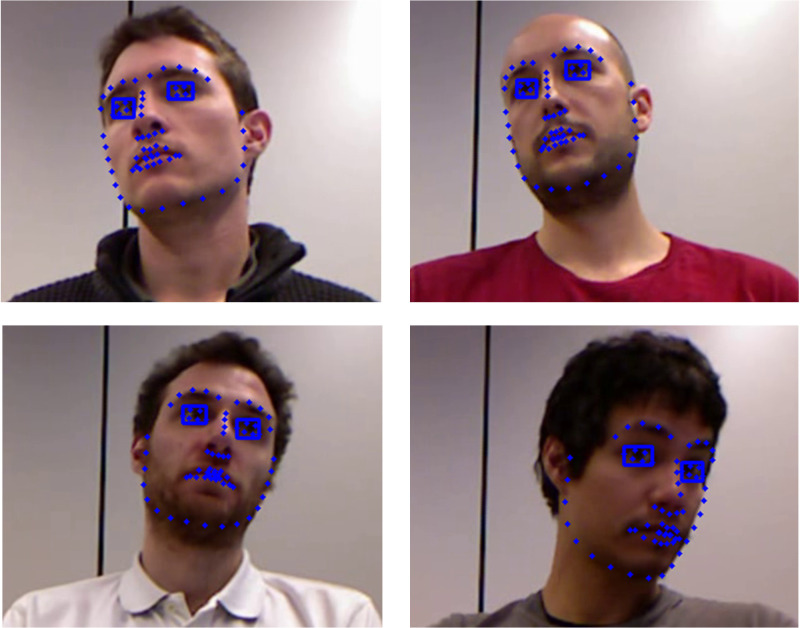
Eye ROIs extraction on the EYEDIAP database

Results in Figure 3 show that accurate eye ROIs can be extracted even when large head rotations occur. Then, the eye ROIs dimensions are magnified by a factor of two. Grayscale erosion with a 1-pixel disk structure element is used in the eye ROIs to delete possible noise. Finally, the simple active contour snakuscule is used to locate the iris centers.

**Figure. 4:**
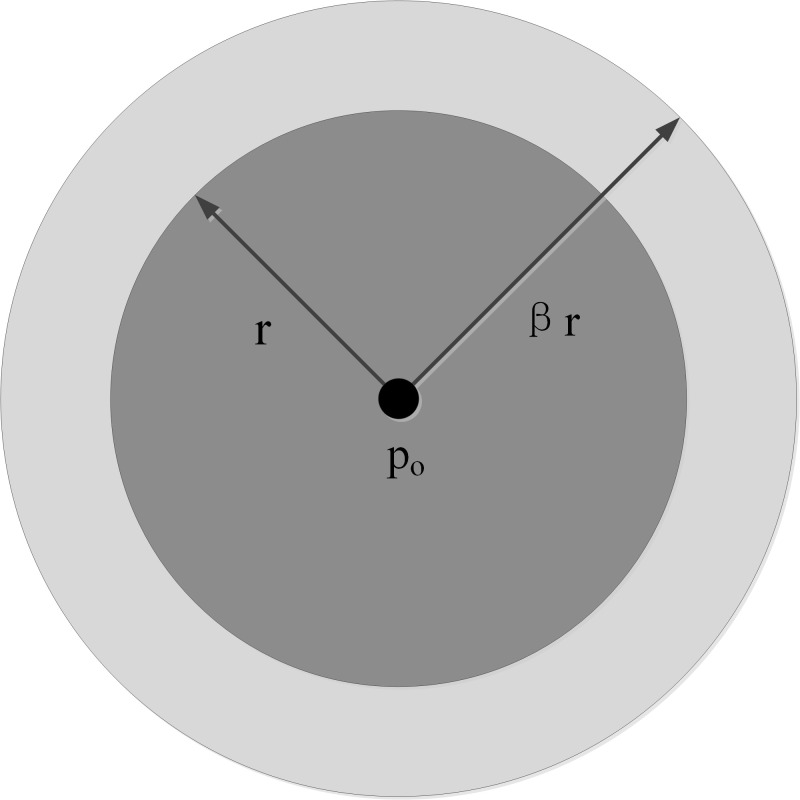
Structure of a snakuscule

As shown in Figure 4, snakuscule is an area-based circular snake that contains an outer annulus and an inner disk. It performs well in detecting circular regions with the maximum gray difference of the outer annulus and the inner disk. *β* (Figure 4) is defined as the ratio of outer to inner radius.

Based on the sclera and the iris having the maximum gray difference, snakuscule can expand or shrink to maximize the values of the outer annulus and the inner disk. However, uncontrolled expansion or shrinkage of the snakuscule needs numerous iterations before its final convergence. To overcome the shortcoming, the snakuscule’s inner radius is initialized by the eye anatomical dimensions that the radius of the eyeball is in the range of 12-13 mm (25) and the radius of the iris is approximately equal to an anatomical constant (approximately 7 mm) (26) for most people. In addition, the method works well as the width of the eye ROI extracted by facial landmarks is close to the diameter of the eyeball in the image. Therefore, the snakuscule inner radius is initialized by

**(1):**
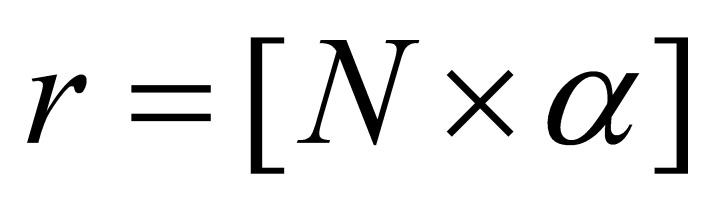


where *N* is the width of the eye ROI and *α* is a constant that involves the ratio of iris radius to the eye ROI width.

Using the initialized snakuscule, the gray difference of the outer annulus and the inner disk is calculated by use of formula (2) is suggested in (12).

**(2):**
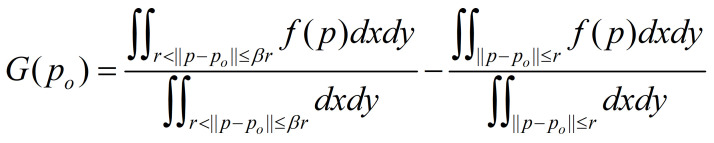


where *f(p)* denotes the image gray value at the position *p*. Formula (2) is used to compute the gray difference of *G(p_i_)*, where *p_i_* = (*x_i_*, *y_i_*), *x_i _*∈ [*βr*, *N*-*βr*], *x_i_* is an integer with a minimum interval of 1, *y_i_* = [*M*/2] and *M* is the height of the eye ROI. In other words, the snakuscule is used to compute gray differences from left to right along the horizontal centerline in the eye ROI. The location *p_rc_*(*x_rc_*, *y_rc_*) with the maximum *G*(*p_i_*) is the rough iris center.

**Figure. 5:**
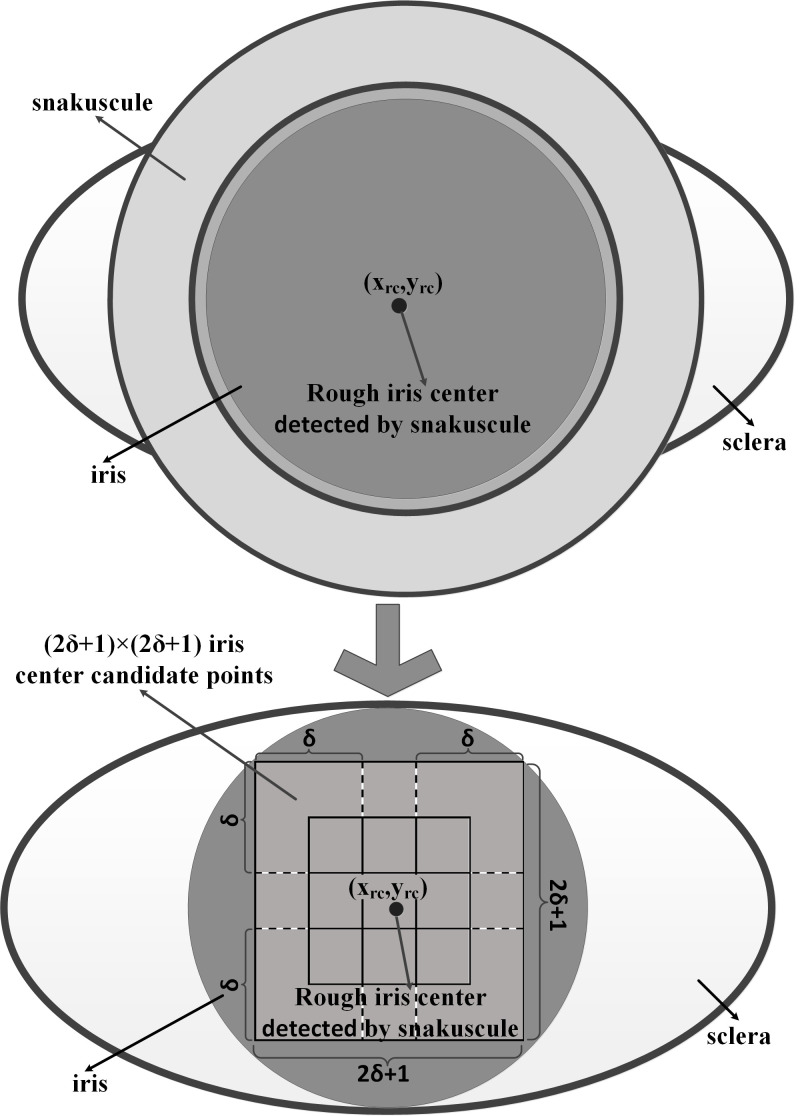
(2δ+1)×(2δ+1) iris center candidate points determined by the rough iris center (x_rc_, y_rc_)

As shown in Figure 5, (2*δ*+1)×(2*δ*+1) iris center candidate points are determined by the rough iris center *(x_rc_, y_rc_)* in the eye ROI. The unit of *δ* is the pixel. The iris center candidate points are used to accurately locate the iris center. In other words, in the range of [*x_rc_*±*δ*, *y_rc_*±*δ*], (2*δ*+1)×(2*δ*+1) gray differences of G were calculated by formula (2). The location *p_c_(x_c_, y_c_)* with the maximum G of the (2*δ*+1)×(2*δ*+1) iris center candidate points was considered as the final iris center.

### Anchor point

Anchor point is used as a reference point to compute the eye vector. The use of the inner or outer eye corners as the anchor points is the common approach (11, 16) among regression-based methods for gaze tracking under natural light. However, Sesma et al. (11) showed that the eye corners vibrate with eye rotations, which introduces errors in the gaze tracking. In (17), the anchor point was set as the center of the image patch tracked by the Lucas–Kanade inverse affine transform. However, sometimes eye corners or the center of the patch cannot be accurately tracked because they may be blocked or deformed in an image with a large head rotation. Therefore, a novel anchor point is designed as the reference point in this paper. The anchor point *p_a_*(*x_a_*, *y_a_*) is computed by

**(3):**
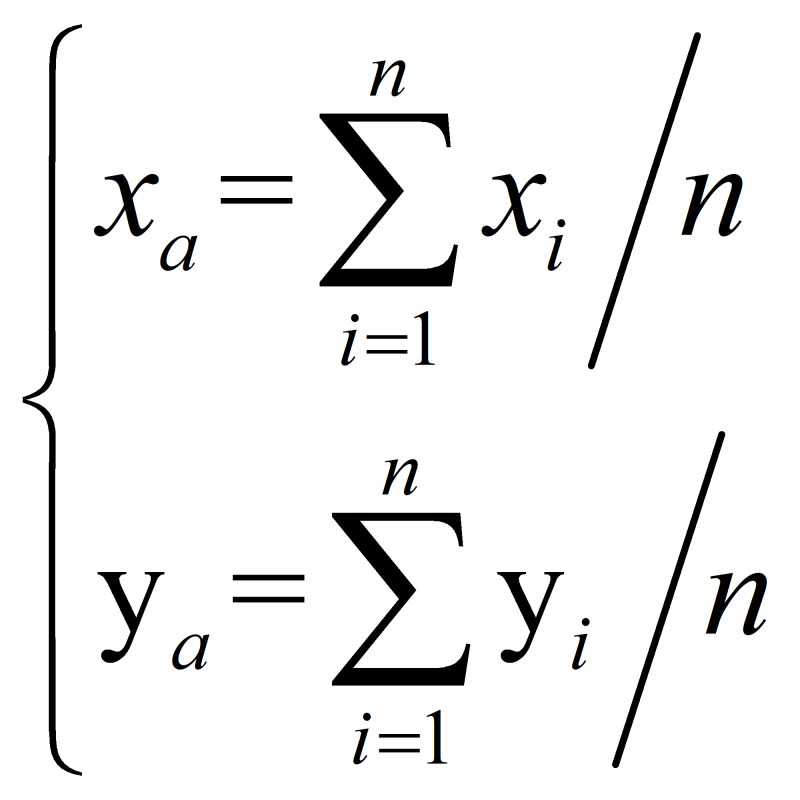


where *n* is the number of facial landmarks and (*x_i_*, *y_i_*) is the coordinate of the *i^th^* facial landmark.

There are two advantages to the anchor point. (1) The anchor point computed by stable facial landmarks does not vibrate with eye rotations and is not blocked with large head movements. (2) The mean of the facial landmarks can reduce the error compared with the single feature point near the eye area.

### Head pose

The action of looking at objects usually involves head movement towards the object, and eye rotation focusing on the object. Fridman et al. (27) used the head pose to track the gaze. However, Kennedy et al. (28) found that gaze tracking merely based on the head pose is neither accurate nor consistent in human-robot interactions. Therefore, gaze tracking should synchronize eye rotation and head movement.

For the past several years, different methods for head pose estimation have been developed. The 2D-3D point correspondence methods achieve robust performance and can address large head movements. Therefore, the OpenCV iterative (Levenberg-Marquardt optimization) algorithm is used to estimate the head pose.

### Mapping functions

After the eye vectors, head pose and screen coordinates have been obtained, the regression strategy is used to establish the mapping function between them. The linear terms, squared terms, cubic terms and interactions summarized in (29) are widely used for mapping eye vectors to screen coordinates. Unlike the head pose that was used to improve the eye vectors in (30), it is directly introduced in the mapping functions. The mapping functions of n points with a polynomial of n or fewer terms can be expressed by

**(4):**
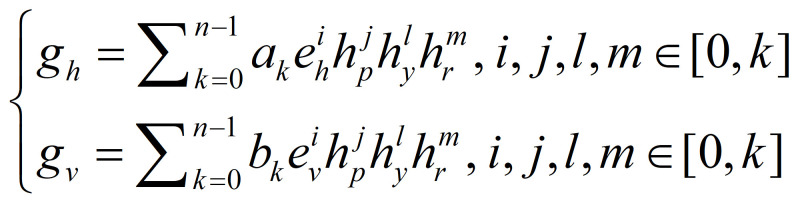


where *g_h_* and *g_v_* are the POR of the horizontal and vertical directions, the coefficients *a_k_* and *b_k_* are determined by the calibration phase, *e_h_* and *e_v_* are the eye vectors of the horizontal and vertical directions, and *h_p_*, *h_y_* and *h_r_* are the head pose angles of the pitch, yaw and roll, respectively. In this paper, six mapping functions derived by formula (4) are used to estimate the gaze. As shown in Table 2, the mapping functions of No.1 and No.2 use the linear and squared terms of eye vectors. No.3, No.4, No.5 and No.6 mix the linear and squared terms of eye vectors and head pose.

**Table 2.**
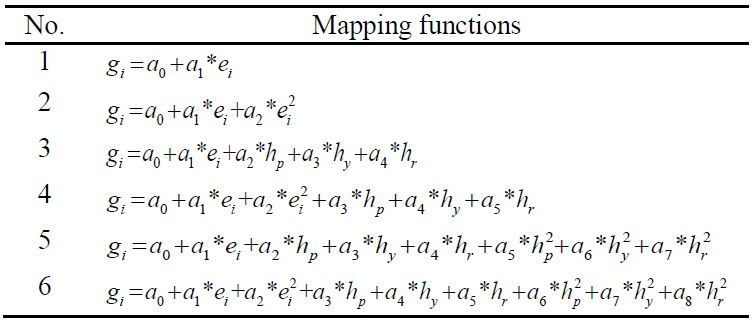
Six mapping functions derived by formula (4), where the subscript “i” denotes “h” or “v”.

Ocular dominance theory is common and long-standing (31). In (32), a dominant eye is shown to be more accurate on SMI HiSpeed 500-Hz eye tracker systems. In addition, Quartley and Firth (33) found that observers favor the left eye for leftward targets and the right eye for rightward targets. Furthermore, for relatively small eye-in-head rotations, Cui and Hondzinski (34) proved that taking the average POR of the two eyes for gaze tracking is more accurate than using only one eye on remote eye tracker. In addition, one of the eyes may be blocked due to a large head movement. To unify these situations, a weight coefficient is used on the POR of the left and right eyes to revise the final POR of the horizontal *g_fh_* and vertical *g_fv_* directions.

**(5):**
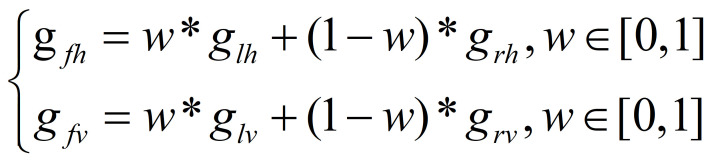


where *w* is the weight coefficient and *g_lh_*, *g_rh_*, *g_lv_* and *g_rv_* are POR of the horizontal and vertical directions of the left and right eyes, respectively.

## Evaluation

### Databases

The GI4E database (35) consists of 1236 images (800×600) from 103 different participants. Each participant has 12 images in which the participant gazed at different points on the screen. A large number of participants with low resolution images make it suitable for evaluating the performance of the proposed iris center localization method.

**Figure. 6:**
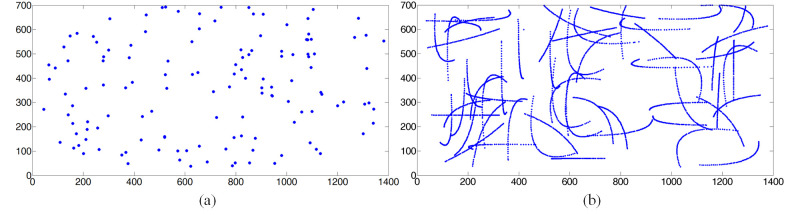
Example of screen coordinates for a video clips using (a) *Discrete Screen target, (b) Continuous Screen target on the EYEDIAP database.*

The EYEDIAP database contains RGB (640×480), RGB-D and HD (1920×1080) video clips from 16 participants. Continuous Screen (CS), Discrete Screen (DS) and 3D Floating Target (FT) are the stimuli that were used for the participants to gaze at. As shown in Figure 6, on the computer screen, DS target was drawn every 1.1 seconds on random locations and CS target was programmed to move along a random trajectory for 2s. The participants were asked to keep an approximately Static (S) or perform head Movements (M) when they gazed at the visual target. Each participant was recorded for 2 to 3 minutes. The proposed method was implemented on the RGB video clips that contains Discrete Screen with Static (DSS) and Discrete Screen with head Movements (DSM), and Continuous Screen with Static (CSS) and Continuous Screen with head Movements (CSM).

On the EYEDIAP database, the frame-by-frame screen target coordinates, head pose tracking states and eyes tracking states including the eyeballs’ 3D coordinates have been provided in the files of "screen_coordinates.txt", "head_pose.txt" and "eyes tracking.txt", respectively. It is noted that, a total of 52 RGB video clips of 13 participants were used to estimate the gaze in this paper because the 12^th^ and 13^th^ participants only recorded the video clips for 3D FT and the 7^th^ participant’s facial landmarks can be tracked on a small fraction of the entire RGB video clips due to the poor contrast.

### Evaluation of iris center localization

The computation of the estimated eye center normalized error by use of formula (6) is suggested in (36).

**(6):**
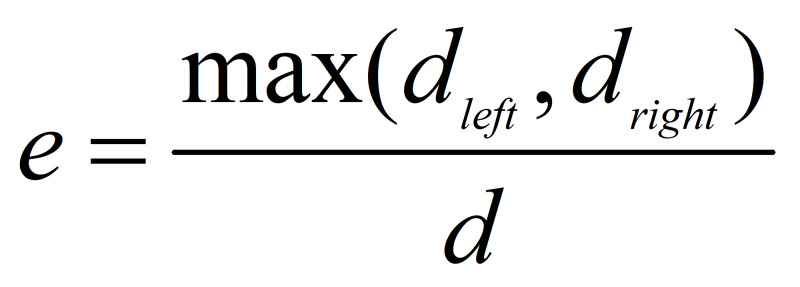


where *d_left_* and *d_right_* are the distances between the estimated and labelled iris centers of the left and right eyes, and *d* is the distance between the labeled left and right iris centers. The estimated eye centers in the range of the normalized error *e* ≤ 0.05 that are equivalent to locate in the pupil can be used for gaze tracking applications (37). Therefore, *e* ≤ 0.05 is used as the benchmark to evaluate the optimal parameters of the iris center localization method in this paper.

For iris center localization, *α*, *β* and *δ* with different values were used on the GI4E database. The optimal values can be obtained when the number of eyes with a normalized error *e* ≤ 0.05 reaches the maximum value. Therefore, values for *α* from 0.21 to 0.25 with the minimum interval of 0.05, *β* from 1.32 to 1.52 with the minimum interval of 0.04 and *δ* from 1 to 4 with the minimum interval of 1 were assessed in this paper

**Figure. 7:**
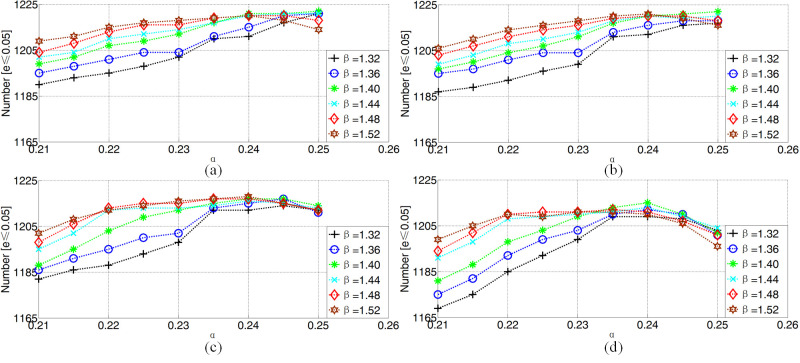
The number of images from the GI4E database with a normalized error e ≤ 0.05 for different values ofα, β and δ, where (a)δ= 1 (b)δ= 2 (c)δ= 3 and (d)δ= 4.

As shown in Figure 7, the maximum number of images with a normalized error *e* ≤ 0.05 is 1222 when *α* = 0.25, *β* = 1.4 and *δ* = 1 or 2.

### Evaluation of different mapping functions

The performance of different mapping functions was compared on the DSS, CSS, DSM and CSM RGB video clips. Iris centers were detected by the optimal parameters of *α* = 0.25, *β* = 1.4 and *δ* = 1 or 2. The anchor point was computed by formula (3), where the unstable facial landmarks around the mouth and eye areas were removed. Therefore, the parameter n equals 36. Then, the eye vectors for the horizontal and vertical directions were computed by the iris centers and the anchor point. Head pose was estimated by the iterative with six points (9, 31, 37, 46, 49 and 55 in Figure 2). Finally, the six mapping functions listed in Table 2 were used to estimate the gaze from the eye vectors and the head pose.

For each RGB video clip on the EYEDIAP database, the first 1000 frames that the faces could be detected were used as calibration frames, and the remaining frames were used as testing frames. The gaze tracking errors for the 13 participants were computed by averaging the results of the participants’ testing frames. The gaze tracking error of each frame is computed by the POR of the left eye, the original 3D coordinate of the eye gaze screen point and the original 3D coordinate of the left eyeball.

The average gaze tracking errors of 52 RGB video clips that were used to evaluate the optimal mapping functions are shown in Table 3. Overall, the mapping functions of No.4 and No.2 achieved the best results in the horizontal and vertical directions, respectively. In addition, the gaze tracking errors show that *δ* = 2 performs better than *δ* = 1. Therefore, the following experimental results that involve the iris centers localization are conducted with *δ* = 2. Meanwhile, the horizontal and vertical gaze tracking errors in the following experiments are regressed by the mapping functions of No.4 and No.2, respectively.

**Table 3.** The average gaze tracking errors (degrees) of 52 RGB video clips on the EYEDIAP database computed by six mapping functions.

No.	*δ* = 1			*δ* = 2		
	H	V	C	H	V	C
1	6.4	4.0	7.5	6.3	3.9	7.4
2	6.4	3.9	7.5	6.2	**3.8**	7.3
3	6.0	4.1	7.3	5.8	4.1	7.1
4	5.8	4.0	7.0	**5.7**	4.0	7.0
5	6.2	4.6	7.7	6.1	4.6	7.6
6	6.1	4.5	7.6	5.9	4.4	7.4

Note: No. denotes the mapping functions in Table 2. H, V and C are the horizontal, vertical and combined gaze tracking errors, respectively. C is the sum of the squares of the H and V. The minimum errors are marked as bold.

### Evaluation of the weight coefficient *w*

**Figure. 8:**
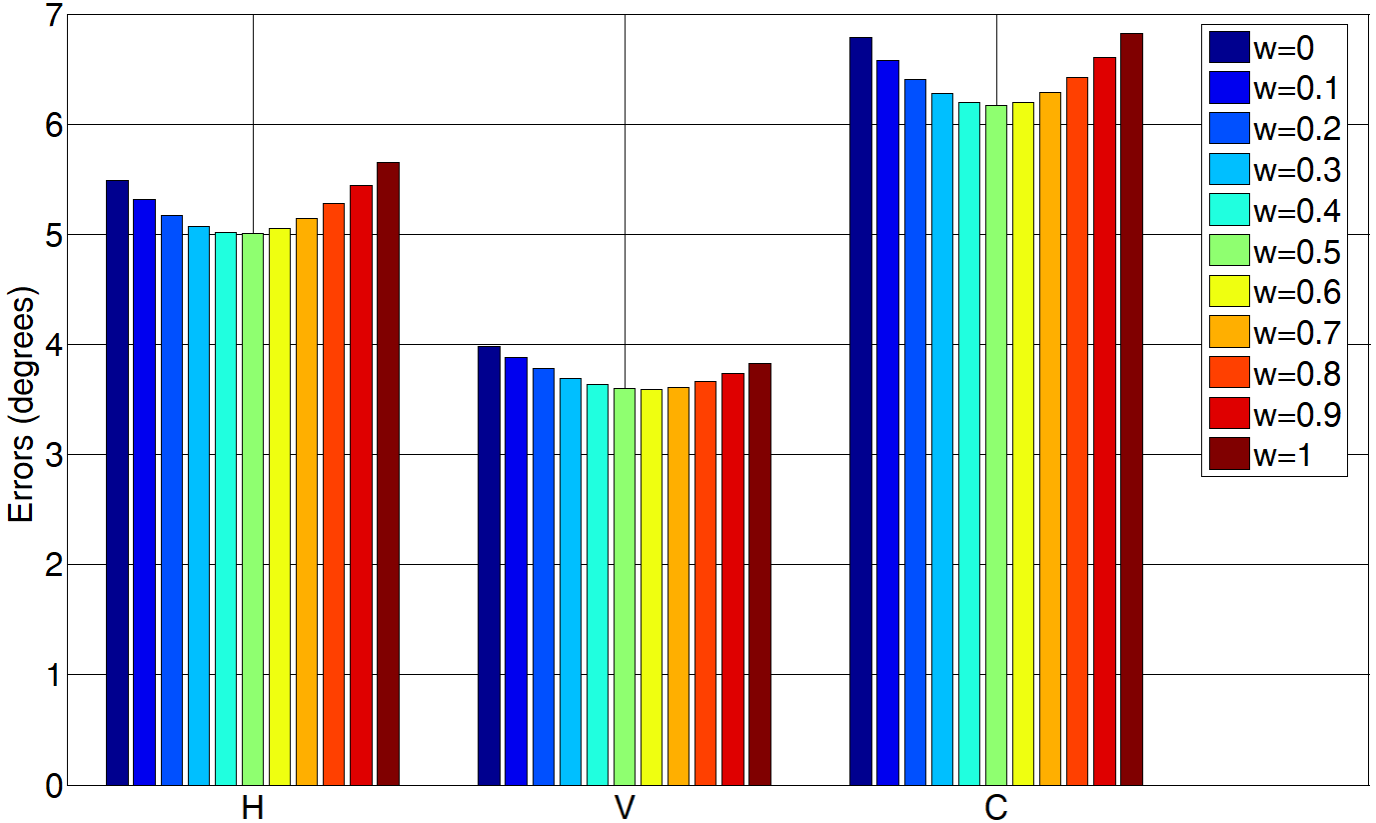
The H, V and C gaze tracking errors *computed by different w on the EYEDIAP database.*

In this paper, the weight coefficient *w* ∈ [0,1] with a minimum value of 0.1 was used to compute the gaze tracking errors on the EYEDIAP database. As shown in Figure 8, *w* = 0.5, 0.6 and 0.5 achieve the best horizontal, vertical and combined gaze tracking errors, respectively. For simplicity, *w* = 0.5 was used in this paper.

**Figure. 9:**
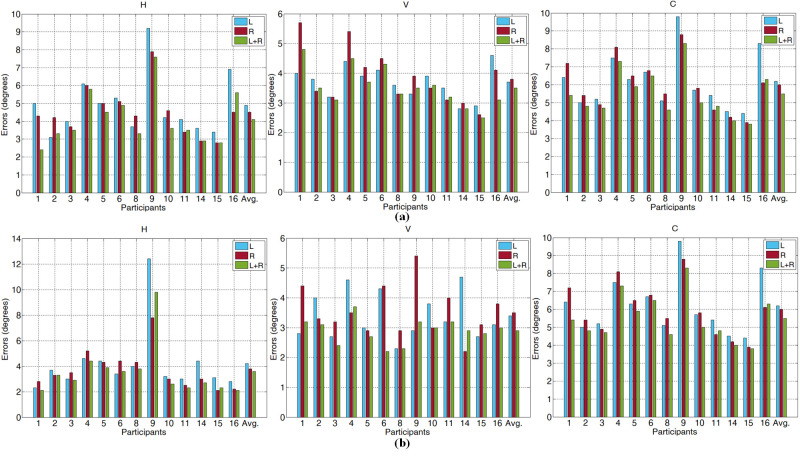
The H, V and C gaze tracking errors on the EYEDIAP (a) DSS and (b) CSS RGB video clips.

To provide more comprehensive improved results by *w* for the 13 participants on the EYEDIAP database, the horizontal, vertical and combined gaze tracking errors on the DSS and CSS RGB video clips are shown in Figure 9. The results on the DSM and CSM RGB video clips are shown in Table 4. L (w = 1), R (w = 0) and L+R (w = 0.5) are the gaze tracking error computed by the POR of the left, right eyes and improved, the original 3D coordinate of the eye gaze screen point and the original 3D coordinate of the left and right eyeball. Meanwhile, the Total Frames (TF) and the Detected Frames (DF) are presented in the table. DF is a frame in which the face can be detected. Compared with the average face detection rate (DF/TF) of 86.9% in (10) on the CSM RGB video clips, an average of 97.2% was obtained in this paper. Considering the low quality frames and large head pose variations in the video clips, we believe the face detection rate is robust. As shown in Figure 9 and Table 4, most of the single eye gaze tracking errors are improved by averaging the POR of both eyes. The method achieved the average combined gaze tracking errors 5.5°, 4.6°, 7.2° and 7.6° on the DSS, CSS, DSM and CSM RGB video clips. Compared with the gaze tracking error of 2.9° under natural light in (17). The reason the errors are high is because that the EYEDIAP database has the lower quality eye image (the iris radius ≈4.5 pixels) compared to (17) self-built database (the iris radius ≈9 pixels).

**Table 4.** The gaze tracking errors (degrees) on the EYEDIAP CSM and DSM RGB video clips, where No. is the participant of the EYEDIAP database.

	DSM											
				H			V			C		
No.	TF	DF	DF/TF	L	R	L+R	L	R	L+R	L	R	L+R
1	4465	3893	87.2%	7.9	8.0	7.7	4.5	4.5	4.5	9.1	9.2	8.9
2	4464	4335	97.1%	7.6	6.5	6.8	3.7	3.8	3.7	8.4	7.6	7.7
3	4433	4322	97.5%	5.9	6.9	5.9	5.7	5.5	5.3	8.2	8.8	7.9
4	4464	4402	98.6%	7.7	7.2	5.7	5.0	4.8	4.5	9.2	8.7	7.3
5	4465	4465	100%	5.9	5.2	4.5	4.5	4.1	4.0	7.4	6.7	6.0
6	4464	4464	100%	5.3	5.5	5.1	4.6	4.6	4.5	7.0	7.2	6.8
8	4465	4020	90.0%	9.2	10.1	8.7	4.8	4.5	4.4	10.4	11.0	9.8
9	4464	4362	97.7%	10.1	6.8	7.5	3.7	3.7	3.6	10.8	7.8	8.3
10	4464	4450	99.7%	9.1	9.5	9.2	4.5	4.8	4.6	10.2	10.7	10.3
11	4465	4465	100%	4.7	3.7	3.5	4.5	6.4	3.5	6.5	7.4	4.9
14	4465	4464	100%	4.7	4.3	3.6	3.7	3.6	3.3	6.0	5.5	4.9
15	4465	4465	100%	3.8	3.4	3.2	4.1	4.2	4.2	5.6	5.4	5.3
16	4465	4286	96.0%	6.1	6.6	4.9	4.0	4.1	3.9	7.3	7.8	6.2
Avg.	4462	4338	97.2%	6.8	6.5	5.9	4.4	4.5	4.2	8.1	7.9	7.2
	CSM											
1	4457	3370	75.6%	9.7	11.2	10.3	4.0	4.1	4.0	10.5	12.0	11.0
2	4457	4360	97.8%	7.9	7.6	7.6	3.2	3.2	3.1	8.5	8.2	8.2
3	4458	3962	88.9%	6.0	7.0	5.9	4.1	3.8	4.0	7.3	8.0	7.1
4	4494	4333	96.4%	8.1	7.0	6.7	3.7	3.8	3.7	8.9	8.0	7.7
5	4458	4394	98.6%	5.3	6.1	5.1	3.8	3.6	3.7	6.5	7.1	6.3
6	4458	4458	100%	7.7	8.4	7.6	4.4	4.5	4.1	8.8	9.6	8.6
8	4458	3510	78.7%	10.6	9.3	9.6	4.1	4.5	4.3	11.4	10.3	10.5
9	4457	4199	94.2%	7.4	7.2	7.2	4.0	3.8	3.8	8.4	8.1	8.1
10	4492	4492	100%	6.5	7.3	6.6	5.0	4.9	4.9	8.2	8.8	8.3
11	4458	4360	97.8%	6.0	6.5	6.2	3.6	3.6	3.6	7.0	7.4	7.1
14	4458	4439	99.6%	4.1	3.3	3.4	3.1	4.1	3.4	5.2	5.2	4.9
15	4458	4458	100%	3.6	3.5	2.9	3.2	3.2	3.1	4.8	4.8	4.2
16	4458	4293	96.3%	5.5	9.7	5.5	4.2	5.8	3.8	6.9	11.3	6.6
Avg.	4463	4202	94.2%	6.8	7.2	6.5	3.9	4.1	3.8	7.9	8.4	7.6

### Computational cost

The method was realized by using the C++ language with Microsoft Visual Studio 2017, OpenCV and the dlib (38) library on a laptop with a 2.7-GHz Intel(R) Core(TM) i7-7500 processor and 8-GB RAM. Data from the EYEDIAP database and the laptop camera were used to measure the execution time, which was computed by averaging the processing time of all testing frames. The execution times of the proposed method are shown in Table 5. It is noted that facial landmarks detection includes face detection and landmarks detection. Experiment results show that face detection consumes most of the processing time. Therefore, the original resolution (640×480) was resized to improve the efficiency of face detection. Facial landmarks are tracked on the faces from the raw frames, in which the faces are obtained by use of the rectangular face ROI detected in the resized frames. Unfortunately, the face detection rate of the EYEDIAP database decreased when the resolution is lower than 512×380 because the RGB video clips have small faces. Therefore, the execution speed is 22 fps for the EYEDIAP database. The mode of data from the laptop camera is closer to the practical system, which had an execution speed of 35 fps. Compared to the IR tracker with an execution speed in excess of 100 fps, natural light trackers still have a long way to go to be usable in practice.

**Table 5.** The execution time of the gaze tracking system.

		Execution time (milliseconds)		
Data	Resolution	Facial landmarks detection	Gaze tracking	fps
EYEDIAP	512×380	44.5	0.7	22
Camera	320×240	27.4	1.2	35

## Discussion

This paper aims to provide a gaze tracking system with a single-camera under natural light to extend its generality. The accuracies of the iris centers and the usability of the anchor point result in more applicable eye vectors. Using the eye vectors and the estimated head pose, second-order polynomial mapping functions are used to compute the POR of the horizontal and vertical directions on the screen. By implementing a weight coefficient on the POR of the left and right eyes, the final gaze errors improved. The iris center localization method has been shown to be accurate on the GI4E database, which consists of low resolution images under realistic conditions of 103 participants. With a normalized error *e* ≤ 0.05, the feature position of the iris center has achieved an error as low as 1.13%.

Compared with the accuracy of 93.92% in (35), the proposed iris center localization method presents a more accurate result of 98.87% for the feature position of the iris center. Moreover, it also outperforms all previous iris center localization methods in the same database. Compared with the average combined gaze tracking errors of 7.2° and 8.9° on the EYEDIAP CSS and CSM RGB video clips in (9), the proposed gaze tracking method reduced the errors by 36% and 14.6%, respectively. Compared with the average gaze tracking errors of 7.6° and 6.7° in horizontal and vertical directions on the EYEDIAP CSM RGB video clips in (10), 1.1° and 2.9°, respectively were reduced by the proposed method. Furthermore, the RGB and RGB-D video clips both were used as inputs in (9, 10).

The gaze tracking errors are significantly better than the appearance-based and the model-based methods, indicating the effectiveness of the regression-based gaze tracking method in low quality images. However, limited by the random gaze trajectories/points on the screen of which the EYEDIAP database is built, 1000 detected frames from the RGB video clips are used in the calibration phase. It is equivalent to the use of approximately 34 seconds from the 2 or 3 minutes of RGB video clips. In a practical application system, the calibration time could be reduced by calibration strategies summarized in (17) and the gaze tracking errors could be reduced by post-calibration regression in (39). In addition, considering the average gaze tracking errors shown in Table 3, the introduction of head pose in the mapping functions does not improve the accuracy of the vertical direction, but reduces the errors of the horizontal direction. The reason is that the eye vectors derived by the iris centers and the anchor point already contain some information of the head pose.

Although the algorithm in (23) presents robustness and accuracy, facial landmarks still cannot be tracked in some images especially on the CSM and DSM RGB video clips. Hence, in future work, the facial landmarks’ algorithm should be improved in low quality images with large head movements. From the results in Figure 9 and Table 4, most of the single eye gaze tracking errors are improved by averaging the POR of both eyes. However, when one eye is blocked due to a large head movement, the *w* of the blocked eye should be decreased or set to 0. Meanwhile, *w* may be affected by the dominant eye that changes with the direction of the gaze (32). Therefore, in the future, a dedicated database with large head pose variations, and various directions of gaze can be built to study choosing a better value of *w*. In addition, the mapping functions are regressed by person-special eye vectors, which results in a person-dependent gaze tracking system. A person-independent gaze tracking system can be researched by normalizing different people’s eye vectors in a feature space.

A gaze tracking method with a non-intrusive sensor under natural light renders the system suitable for universal use on smartphones, laptops or tablets with a camera. The system, with an accuracy of approximately 6°, can be used in secure authentication of biometrics (40) and gaze-based password entry fields for reducing shoulder-surfing (41). The proposed gaze tracking method further bridges the interaction gap between humans and machines.

## Ethics and Conflict of Interest

The author(s) declare(s) that the contents of the article are in agreement with the ethics described in http://biblio.unibe.ch/portale/elibrary/BOP/jemr/ethics.html and that there is no conflict of interest regarding the publication of this paper.
